# Supplementary material for: Nationwide COVID-19-EII Study: Incidence, Environmental Risk Factors and Long-Term Follow-Up of Patients with Inflammatory Bowel Disease and COVID-19 of the ENEIDA Registry
Source: J Clin Med. 2022 Jan 14;11(2):421. doi: 10.3390/jcm11020421 (PMC8781643; doi:10.3390/jcm11020421)
Supplement: Supplementary file 1 [file jcm-11-00421-s001.zip › jcm-1524992-supplementary.pdf]

Supplementary Table S1: Comorbidities

**Comorbidities**	
Arterial hypertension, *n* (%)	106 (22)
Dyslipidaemia, *n* (%)	74 (15)
Immune-mediated diseases, *n* (%)	53 (11)
Diabetes mellitus, *n* (%)	35 (7.2)
Ischemic cardiomyopathy, *n* (%)	30 (6.2)
Chronic renal insufficiency, *n* (%)	20 (4.1)
Chronic Obstructive Pulmonary Disease, *n* (%)	18 (3.7)
Neoplasia, *n* (%)	17 (3.5)
Cirrhosis, *n* (%)	6 (1.2)
Cerebral stroke, *n* (%)	5 (1)
Dementia, *n* (%)	4 (0.8)
Comorbidities, *n* (%)	
None	272 (56)
One	111 (23)
Two	52 (11)
Three or more	49 (10)
Charlson score, *n* (%)	
Zero	174 (36)
Mild (one-two)	196 (40)
Moderate (three-four)	66 (14)
Severe (five or more)	48 (9.9)

Supplementary Table S2. Symptoms at COVID-19 diagnosis

**Symptoms at COVID-19 diagnosis**	
Fever, *n* (%)	336 (69)
Cough, *n* (%)	305 (63)
Asthenia, *n* (%)	184 (38)
Myalgia/Arthralgia, *n* (%)	148 (30)
Dyspnoea, *n* (%)	134 (28)
Diarrhoea, *n* (%)	126 (26)<br><br>IBD in remission 100 vs. Active IBD 26, p=0.1
Anosmia, *n* (%)	117 (24)
Headache, *n* (%)	113 (23)
Dysgeusia, *n* (%)	81 (17)
Expectoration, *n* (%)	34 (7)
Abdominal pain, *n* (%)	32 (6.6)
Nasal congestion, *n* (%)	27 (5.6)
Odynophagia, *n* (%)	17 (3.5)
Low-grade fever, *n* (%)	12 (2.5)
Nausea/vomiting, *n* (%)	7 (1.4)
Cutaneous lesions, *n* (%)	8 (1.6)
Chest pain, *n* (%)	7 (1.4)

Supplementary Table S3. Tests performed for COVID-19 diagnosis

SARS-CoV-2 PCR, *n* (%)	388 (80)
Positive	301 (62)
Negative	86 (18)
Number of previous negative PCR before testing positive	
One	66 (14)
Two	13 (2.7)
Three or more	6 (1.2)
SARS-CoV-2 serology, *n* (%)	167 (35)
IgG +	113 (23)
IgM +	32 (6.6)
Point of care +	22 (4.6)
No PCR nor SARS-CoV-2 serology, *n* (%)	49 (10.2)
PCR and/or SARS-CoV-2 serology negative, *n* (%)	39 (8.1)
Chest-X-ray, *n* (%)	292 (61)
Abnormal	173 (36)
Unilateral disease	46 (9.5)
Bilateral disease	124 (26)
Lung consolidation	78 (16)
Interstitial disease	49 (10)
Ground glass appearance	44 (9)

Supplementary Table S4. Clinical characteristics and treatment for IBD in patients who required **hospitalisation**

**Variable**	**Hospitalised** *n* **= 168**	**Non hospitalised** *n* **= 314**	**Univariateanalysis**	**Multivariate analysis**		
			***P*-value**	**OR**	**CI**	***P-value***
Male gender, *n* (%)	102 (61)	149 (47)	0.005	1.37	0.88-2.1	0.16
Ulcerative colitis, *n* (%)	86 (51)	135 (43)	0.09	0.8	0.49-1.3	0.39
E1	16 (9.5)	27 (8.6)				
E2	36 (21)	44 (14)				
E3	34 (20)	64 (47)				
Crohn's disease location, *n* (%)			0.17			
L1	42 (25)	71 (23)				
L2	15 (8.9)	28 (8.9)				
L3	21 (12)	68 (22)				
L4 (isolated)	1 (0.6)	2 (0.6)				
Crohn's disease behaviour, *n* (%)						
B1	46 (27)	98 (31)	0.88			
B2	19 (11)	52 (17)	0.30			
B3	18 (10.7)	29 (9.2)	0.27			
Perianal disease, *n* (%)	19 (11)	40 (13)	0.87			
Extraintestinal manifestations, *n* (%)	47 (28)	78 (25)	0.14			
Active smoker, *n* (%)	18 (10.7)	35 (11)	0.99			
≥ 40 years, *n* (%)	149 (89)	237 (75)	0.001			
≥ 50 years, *n* (%)	128 (76)	160 (51)	**< 0.0001**	**2.09**	**1.3-3.4**	**0.004**
≥ 60 years, *n* (%)	84 (50)	65 (21)	< 0.0001			
Family history of IBD, *n* (%)	17 (10)	47 (15)	0.33			
≥1 comorbidity, *n* (%)	105 (62)	106 (34)	**< 0.0001**	**2.28**	**1.4-3.6**	**0.001**
≥2 comorbidity, *n* (%)	60 (36)	40 (13)	< 0.0001			
Charlson score ≥1, *n* (%)	132 (78)	177 (56)	< 0.0001			
Charlson score ≥2, *n* (%)	102 (61)	87 (28)	< 0.0001			
Occupational risk, *n* (%)	30 (18)	103 (33)	**0.001**	0.71	0.4-1.2	0.19
Good social distancing, *n* (%)	89 (53)	146 (46)	0.05	1.3	0.8-2.05	0.26
Active IBD, *n* (%)	27 (16)	56 (18)	0.98			

Steroids 3 months before COVID-19, *n* (%)	24 (14)	29 (9.2)	**0.09**	**1.3**	**1.1-1.6**	**0.03**
5-aminosalycilates, at COVID-19 diagnosis, *n* (%)	84 (50)	124 (39)	0.03	0.96	0.5-1.8	0.89
Steroids at COVID-19 diagnosis, *n* (%)	11 (6.5)	15 (4.7)	0.41			
Immunomodulators at COVID-19 diagnosis, *n* (%)	56 (33)	115 (37)	0.47			
Tiopurines, *n* (%)	45 (27)	102 (61)				
Methotrexate, *n* (%)	8 (4.8)	11 (3.5)				
Biologics at COVID-19 diagnosis, *n* (%)	50 (30)	127 (40)	0.06	0.96	0.5-1.8	0.89
Anti-TNF at COVID-19 diagnosis, *n* (%)	28 (17)	89 (28)	0.004	0.6	0.3-1.3	0.19

IBD: inflammatory bowel disease, L1: ileal, L2: colonic, L3: ileocolic, L4: upper gastrointestinal tract. B1: inflammatory, B2: stricturing, B3: penetrant. E1: proctocolitis, E2: left-sided colitis, E3: pancolitis, TNF: tumour necrosis factor

Supplementary Table S5. Clinical characteristics and treatment for IBD in patients who required **ICU admission**

**Variable**	**ICU admission**<br>***n* = 13**	**Non-ICU admission**<br>***n* = 469**	**Univariate analysis**	**Multivariate analysis**		
			***P*-value**	**OR**	**CI**	***P-value***
Male gender, *n* (%)	9 (69)	224 (48)	0.18			
Ulcerative colitis, *n* (%)<br>E1<br>E2<br>E3	9 (69)<br>1 (7.7)<br>3 (23)<br>5 (38)	201 (43)<br>40 (8.5)<br>72 (15)<br>89 (19)	0.11			
Crohn's disease location, *n*<br>(%)<br>L1<br>L2<br>L3<br>L4 (isolated)	 2 (15)<br>0<br>2 (15)<br>0	 104 (22)<br>41 (8.7)<br>82 (17)<br>3 (0.6)	0.89			
Crohn's disease behaviour,<br>*n* (%)<br>B1<br>B2<br>B3	 1 (7.7)<br>2 (15)<br>2 (15)	 140 (30)<br>63 (13)<br>43 (9.1)	 0.13<br>1<br>0.83			
Perianal disease, *n* (%)	2 (15)	55 (12)	0.54			
Extraintestinal manifestations, *n* (%)	3 (23)	119 (25)	0.84			
Active smoker, *n* (%)	0	49 (10)	0.26			
≥ 40 years, *n* (%)	11 (85)	357 (76)	0.94			
≥ 50 years, *n* (%)	11 (85)	264 (56)	0.07			
≥ 60 years, *n* (%)	6 (46)	133 (28)	0.34			
Family history of IBD, *n* (%)	3 (23)	57 (12)	0.53			
≥1 comorbidity, *n* (%)	8 (61)	191 (41)	0.18			
≥2 comorbidity, *n* (%)	5 (38)	90 (19)	0.21			
Charlson score ≥1, *n* (%)	13 (100)	308 (60)	0.02			
Charlson score ≥2, *n* (%)	10 (77)	169 (36)	**0.005**	**5.4**	**1.5-20.1**	**0.011**

Occupational risk, *n* (%)	3 (23)	126 (27)	1			
Good social distancing, *n* (%)	9 (69)	218 (46)	0.21			
Active IBD, *n* (%)	6 (46)	101 (21)	0.10			
Abnormal chest X-ray, *n* (%)	12 (92)	156 (33)	0.01			
Steroids 3 months before COVID-19, *n* (%)	1 (7.7)	50 (10.7)	1			
5-aminosalycilates, at COVID-19 diagnosis, *n* (%)	10 (77)	187 (40)	**0.012**	**Unadjusted**		
				**4.6**	**1.2-17**	**0.02**
				**Adjusted to ulcerative colitis**		
				3.6	0.85-15.2	0.08
Steroids at COVID-19 diagnosis, *n* (%)	1 (7.7)	24 (5)	1			
Immunomodulators at COVID-19 diagnosis, *n* (%)	3 (23)	159 (34)	0.5			
Tiopurines, *n* (%)	3 (23)	136 (29)				
Methotrexate, *n* (%)	0	19 (4)				
Biologics at COVID-19 diagnosis, *n* (%)	4 (31)	164 (35)	0.88			
Anti-TNF at COVID-19 diagnosis, *n* (%)	3 (23)	108 (23)	1			

IBD: inflammatory bowel disease, L1: ileal, L2: colonic, L3: ileocolic, L4: upper gastrointestinal tract. B1: inflammatory, B2: stricturing, B3: penetrant. E1: proctocolitis, E2: left-sided colitis, E3: pancolitis, TNF: tumour necrosis factor

Supplementary Table S6. Clinical characteristics and treatment for IBD in patients who **died due to COVID-19**

Variable	Death due to COVID-19 *n* = 18	Alive *n* = 462	Univariate analysis	Multivariate analysis		
			P-value	OR	CI	*P-value*
Male gender, *n* (%)	12 (67)	238 (51)	0.21			
Ulcerative colitis, *n* (%)	7 (39)	213 (46)	0.48			
E1	1 (5.6)	42 (9)				
E2	4 (22)	75 (16)				
E3	2 (11)	96 (21)				
Crohn's disease location, *n* (%)			0.95			
L1	6 (33)	107 (23)				
L2	1 (5.5)	42 (9)				
L3	4 (22)	84 (18)				
L4 (isolated)	0	3 (0.6)				
Crohn's disease behaviour, *n* (%)						
B1	4 (22)	140 (30)	0.23			
B2	3 (17)	68 (15)	1			
B3	5 (28)	42 (9)	0.06			
Active B3	2 (11)	10 (2.2)	0.17			
Perianal disease, *n* (%)	5 (28)	54 (12)	0.18			
Extraintestinal manifestations, *n* (%)	4 (22)	121 (26)	0.78			
Active smoker, *n* (%)	2 (11)	51 (11)	0.99			
≥ 40 years, *n* (%)	16 (89)	368 (80)	0.50			
≥ 50 years, *n* (%)	16 (89)	270 (58)	0.01			
≥ 60 years, *n* (%)	15 (83)	132 (28)	**< 0.0001**	**7.1**	**1.8-27.4**	**0.004**
Family history of IBD, *n* (%)	3 (17)	60 (13)	0.17			
≥1 comorbidity, *n* (%)	16 (89)	193 (42)	< 0.0001			
≥2 comorbidity, *n* (%)	12 (67)	87 (19)	**< 0.0001**	**3.9**	**1.3-11.6**	**0.01**
Charlson score ≥1, *n* (%)	18 (100)	289 (62)	0.001			
Charlson score ≥2, *n* (%)	17 (94)	170 (37)	< 0.0001			
Occupational risk, *n* (%)	2 (11)	131 (28)	0.28			
Good social distancing, *n* (%)	12 (67)	222 (48)	0.03			

Active IBD, *n* (%)	5 (28)	91 (20)	0.59	
Abnormal chest X-ray, *n* (%)	16 (89)	156 (34)	0.001	
Steroids 3 months before COVID-19, *n* (%)	1 (5.5)	51 (11)	0.73	
5-aminosalycilates, at COVID-19 diagnosis, *n* (%)	10 (55)	197 (43)	0.28	
Steroids at COVID-19 diagnosis, *n* (%)	1 (5.5)	24 (5.2)	1	
Immunomodulators at COVID-19 diagnosis, *n* (%)	4 (22)	166 (36)	0.23	
Tiopurines, *n* (%)	3 (17)	143 (31)		
Methotrexate, *n* (%)	1 (5.5)	18 (3.9)		
Biologics at COVID-19 diagnosis, *n* (%)	7 (39)	170 (37)	0.86	
Anti-TNF at COVID-19 diagnosis, *n* (%)	4 (22)	113 (24)	1	

IBD: inflammatory bowel disease, L1: ileal, L2: colonic, L3: ileocolic, L4: upper gastrointestinal tract. B1: inflammatory, B2: stricturing, B3: penetrant. E1: proctocolitis, E2: left-sided colitis, E3: pancolitis, TNF: tumour necrosis factor

Supplementary Table S7. Clinical characteristics and treatment for IBD in patients with **Severe COVID-19***

**Variable**	**Severe COVID-19** *n* **= 38**	**Not severe COVID-19** *n* **= 444**	**Univariate analysis**	**Multivariate analysis**		
			P-value	**OR**	**CI**	***P-value***
Male gender, *n* (%)	24 (63)	227 (51)	0.15			
Ulcerative colitis, *n* (%)	19 (50)	202 (46)	0.60			
E1	4 (10.5)	39 (8.8)				
E2	9 (24)	71 (16)				
E3	6 (16)	92 (20)				
Crohn's disease location, *n* (%)			0.65			
L1	11 (29)	102 (23)				
L2	2 (5.3)	41 (9.1)				
L3	5 (13)	83 (19)				
L4 (isolated)	0	3 (0.7)				
Crohn's disease behaviour, *n* (%)						
B1	6 (16)	138 (31)	**0.03**	**0.29**	**0.1-0.9**	**0.03**
Active B1	2 (5.3)	31 (6.9)	1			
B2	7 (18)	64 (14)	0.32			
B3	6 (16)	41 (9.1)	0.19			
Perianal disease, *n* (%)	5 (13)	54 (12)	0.92			
Extraintestinal manifestations, *n* (%)	9 (24)	116 (26)	0.80			
Active smoker, *n* (%)	3 (7.9)	50 (11)	0.88			
Abnormal chest X-ray, *n* (%)	33 (87)	140 (32)	<0.0001			
≥ 40 years, *n* (%)	34 (89)	352 (77)	0.13			
≥ 50 years, *n* (%)	33 (87)	255 (57)	<0.0001			
≥ 60 years, *n* (%)	26 (68)	123 (28)	**<0.0001**	**4.59**	**1.3-15.9**	**0.02**
Family history of IBD, *n* (%)	8 (21)	56 (13)	0.35			
≥1 comorbidity, *n* (%)	30 (79)	181 (41)	**<0.0001**	3.25	0.94-11.3	0.06
≥2 comorbidity, *n* (%)	19 (50)	82 (18)	<0.0001			

Charlson score ≥1, *n* (%)	36 (95)	273 (61)	<0.0001			
Charlson score ≥2, *n* (%)	32 (84)	157 (35)	<0.0001			
Occupational risk, *n* (%)	7 (18)	126 (28)	0.26			
Good social distancing, *n* (%)	25 (66)	210 (47)	0.01	3.47	0.89-13.45	0.07
Active IBD, *n* (%)	11 (29)	101 (23)	0.38			
Steroids 3 months before COVID-19, *n* (%)	5 (13)	48 (10.8)	0.86			
5-aminosalycilates, at COVID-19 diagnosis, *n* (%)	25 (66)	183 (41)	**0.003**	2.2	0.67-7.33	0.19
Steroids at COVID-19 diagnosis, *n* (%)	4 (10.5)	22 (4.9)	0.28			
Immunomodulators at COVID-19 diagnosis, *n* (%)	8 (21)	163 (37)	**0.05**	0.676	0.2-2.1	0.51
Tiopurines, *n* (%)	7 (18)	140 (31)				
Methotrexate, *n* (%)	1 (2.6)	18 (4)				
Biologics at COVID-19 diagnosis, *n* (%)	12 (32)	165 (37)	0.49			
Anti-TNF at COVID-19 diagnosis, *n* (%)	7 (18)	110 (25)	0.38			

IBD: inflammatory bowel disease, L1: ileal, L2: colonic, L3: ileocolic, L4: upper gastrointestinal tract. B1: inflammatory, B2: stricturing, B3: penetrant. E1: proctocolitis, E2: left-sided colitis, E3: pancolitis, TNF: tumour necrosis factor

*Severe COVID-19: composite of intensive care unit admission and/or use of active amines and/or respiratory distress and/or invasive oxygen therapy and/or death

Supplementary Table S8. Incidence of adverse outcomes of COVID-19 patient in the ENEIDA registry

**Hospitalisation**	
< 50 years: 160 x 100 000<br><br>≥ 50 years: 475x 100 000	Men: 386 x 100 000<br><br>Women: 260 x 100 000
**Death**	
< 50 years: 8 x 100 000<br><br>≥ 50 years: 59 x 100 000	Men: 38 x 100 000<br><br>Women: 24 x 100 000
**ICU admission**	
< 50 years: 8 x 100 000<br><br>≥ 50 years: 41 x 100 000	Men: 34 x 100 000<br><br>Women: 16 x 100 000
**Severe COVID-19***	
< 50 years: 21 x 100 000<br><br>≥ 50 years: 122 x 100 000	Men: 91 x 100 000<br><br>Women: 56 x 100 000

*Severe COVID-19: composite of intensive care unit admission and/or use of active amines and/or respiratory distress and/or invasive oxygen therapy and/or death

Supplementary Table S9. Clinical characteristics and treatment for IBD in patients with **3 months physical sequelae**

**Variable**	**Physical sequelae at 3 months** *n* **= 56**	**Not physical sequelae at 3 months** *n* **= 406**	**Univariate analysis**	**Multivariate analysis**		
			***P*-value**	**OR**	**CI**	***P-value***
Male gender, *n* (%)	29 (52)	209 (51)	0.97			
Ulcerative colitis, *n* (%)	28 (50)	185 (45)	0.32			
E1	3 (5.4)	36 (8.9)				
E2	16 (29)	59 (15)				
E3	12 (21)	82 (20)				
Crohn's disease location, *n* (%)			0.81			
L1	9 (16)	98 (24)				
L2	4 (7.1)	38 (9.4)				
L3	11 (20)	73 (18)				
L4 (isolated)	0	3 (0.7)				
Crohn's disease behaviour, *n* (%)						
B1	15 (27)	125 (31)	0.74			
B2	7 (13)	61 (15)	0.97			
B3	3 (5.4)	39 (9.6)	0.66			
Perianal disease, *n* (%)	6 (11)	49 (12)	0.24			
Extraintestinal manifestations, *n* (%)	16 (29)	105 (26)	0.56			
Active smoker, *n* (%)	7 (13)	44 (11)	0.95			
Abnormal chest X-ray, *n* (%)	23 (41)	133 (33)	0.25			
≥ 40 years, *n* (%)	49 (88)	319 (79)	0.12			
≥ 50 years, *n* (%)	41 (73)	229 (56)	0.02	1.3	0.3-5.9	0.71
≥ 60 years, *n* (%)	18 (32)	114 (28)	0.53			
Family history of IBD, *n* (%)	3 (5.3)	57 (14)	0.19			
≥1 comorbidity, *n* (%)	29 (52)	164 (40)	0.11			
≥2 comorbidity, *n* (%)	15 (27)	72 (18)	0.10			
Charlson score ≥1, *n* (%)	43 (77)	246 (60)	0.02	1.5	0.32-6.9	0.62
Charlson score ≥2, *n* (%)	26 (46)	144 (35)	0.11			
Occupational risk, *n* (%)	13 (23)	118 (29)	0.48			
Good social distancing, *n* (%)	26 (46)	188 (46)	0.79			

Active IBD, *n* (%)	12 (21)	71 (17)	0.49			
Steroids 3 months before COVID-19, *n* (%)	12 (21)	39 (9.6)	**0.008**	**1.4**	**1.07-1.7**	**0.01**
5-aminosalycilates, at COVID-19 diagnosis, *n* (%)	32 (57)	165(41)	0.019	1.7	0.98-3.1	0.06
Steroids at COVID-19 diagnosis, *n* (%)	6 (10.7)	18 (4.4)	0.1			
Immunomodulators at COVID-19 diagnosis, *n* (%)	17 (30)	149 (37)	0.35			
Tiopurines, *n* (%)	16 (29)	127 (31)				
Methotrexate, *n* (%)	1 (1.8)	17 (4.2)				
Biologics at COVID-19 diagnosis, *n* (%)	17 (30)	153 (38)	0.29			
Anti-TNF at COVID-19 diagnosis, *n* (%)	16 (29)	97 (24)	0.44			
Hospitalisation, *n* (%)	26 (46)	124 (30)	0.02	1.57	0.87-2.8	0.14
ICU admission, *n* (%)	3 (5.3)	5 (1.2)	0.11			

IBD: inflammatory bowel disease, ICU: intensive care unit, L1: ileal, L2: colonic, L3: ileocolic, L4: upper gastrointestinal tract. B1: inflammatory, B2: stricturing, B3: penetrant. E1: proctocolitis, E2: left-sided colitis, E3: pancolitis, TNF: tumour necrosis factor

Supplementary Table S10. Clinical characteristics and treatment for IBD in patients with **12 months physical sequelae**

Variable	Physical sequelae at 12 months *n* = 67	Not physical sequelae at 12 months *n* = 385	Univariate analysis	Multivariate analysis		
			P-value	OR	CI	*P-value*
Male gender, *n* (%)	36 (54)	197 (51)	0.7			
Ulcerative colitis, *n* (%)	31 (46)	177 (46)	0.86			
E1	5 (7.5)	37 (9.6)				
E2	12 (18)	63 (16)				
E3	11 (16)	85 (22)				
Crohn's disease location, *n* (%)			0.72			
L1	11 (16)	69 (18)				
L2	8 (12)	31 (8.1)				
L3	8 (12)	62 (16)				
L4 (isolated)	0	3 (0.8)				
Crohn's disease behaviour, *n* (%)						
B1	20 (30)	116 (30)	0.83			
B2	9 (13)	58 (15)	0.81			
B3	4 (5.9)	37 (9.6)	0.36			
Perianal disease, *n* (%)	15 (22)	56 (15)	0.1			
Extraintestinal manifestations, *n* (%)	24 (36)	94 (24)	0.15			
Active smoker, *n* (%)	6 (8.9)	45 (12)	0.13			
Abnormal chest X-ray, *n* (%)	32 (48)	124 (32)	0.08	1.8	0.2-5.8	0.17
≥ 40 years, *n* (%)	58 (87)	302 (78)	0.13			
≥ 50 years, *n* (%)	49 (73)	214 (56)	0.07	1	0.2-4.8	0.95
≥ 60 years, *n* (%)	20 (30)	107 (28)	0.73			
Family history of IBD, *n* (%)	7 (10)	50 (13)	0.83			
≥1 comorbidity, *n* (%)	29 (43)	160 (42)	0.79			
≥2 comorbidity, *n* (%)	18 (27)	67 (17)	0.07			
Charlson score ≥1, *n* (%)	51 (76)	231 (60)	0.01	1.2	0.2.5.8	0.84
Charlson score ≥2, *n* (%)	30 (45)	134 (35)	0.12			
Occupational risk, *n* (%)	17 (25)	111 (29)	0.63			
Good social distancing, *n* (%)	34 (51)	177 (46)	0.68			

Active IBD, *n* (%)	12 (18)	70 (18)	0.93			
Steroids 3 months before COVID-19, *n* (%)	11 (16)	40 (10)	0.15			
5-aminosalycilates, at COVID-19 diagnosis, *n* (%)	34 (51)	159 (41)	0.15			
Steroids at COVID-19 diagnosis, *n* (%)	6 (8.9)	18 (4.7)	0.25			
Immunomodulators at COVID-19 diagnosis, *n* (%)	22 (33)	143 (37)	0.5			
Tiopurines, *n* (%)	17 (25)	125 (32)				
Methotrexate, *n* (%)	5 (7.5)	13 (3.4)				
Biologics at COVID-19 diagnosis, *n* (%)	23 (34)	145 (38)	0.6			
Anti-TNF at COVID-19 diagnosis, *n* (%)	15 (22)	96 (25)	0.66			
Hospitalisation, *n* (%)	30 (45)	119 (31)	0.03	0.95	0.4-2.2	0.89
ICU admission, *n* (%)	2 (2.9)	6 (1.6)	0.75			

IBD: inflammatory bowel disease, ICU: intensive care unit, L1: ileal, L2: colonic, L3: ileocolic, L4: upper gastrointestinal tract. B1: inflammatory, B2: stricturing, B3: penetrant. E1: proctocolitis, E2: left-sided colitis, E3: pancolitis, TNF: tumour necrosis factor
